# Supplementary material for: Strong bulk photovoltaic effect in engineered edge-embedded van der Waals structures
Source: Nat Commun. 2023 Jul 15;14:4230. doi: 10.1038/s41467-023-39995-0 (PMC10349808; doi:10.1038/s41467-023-39995-0)
Supplement: Supplementary file 1 — Supplementary Information [file 41467_2023_39995_MOESM1_ESM.pdf]

Supplementary Information for "Strong bulk photovoltaic effect in engineered edge-embedded van der Waals structures"

Zihan Liang[1,†], Xin Zhou[2,†], Le Zhang[1,†], Xiang-Long Yu[3,4]*, Yan Lv[5], Xuefen Song[5], Yongheng Zhou[1], Han Wang[1], Shuo Wang[5], Taihong Wang[1], Perry Ping Shum[1], Qian He[2], Yanjun Liu[1], Chao Zhu[6], Lin Wang[5,]*, Xiaolong Chen[1,]*

[1] Department of Electrical and Electronic Engineering, Southern University of Science and Technology, 1088 Xueyuan Avenue, Shenzhen 518055, China.

[2] Department of Materials Science and Engineering, National University of Singapore, Singapore 117575, Singapore.

[3] Shenzhen Institute for Quantum Science and Engineering, Southern University of Science and Technology, 1088 Xueyuan Avenue, Shenzhen 518055, China.

[4] International Quantum Academy, Shenzhen 518048, China.

[5] School of Flexible Electronics (Future Technologies) & Institute of Advanced Materials (IAM), Key Laboratory of Flexible Electronics (KLOFE), Jiangsu National Synergetic Innovation Center for Advanced Materials (SICAM), Nanjing Tech University (NanjingTech), Nanjing 211816, China.

[6] SEU-FEI Nano-Pico Center, Key Laboratory of MEMS of Ministry of Education, Collaborative Innovation Center for Micro/Nano Fabrication, Device and System, Southeast University, Nanjing 210096, China.

[†] These authors contributed equally: Zihan Liang, Xin Zhou, and Le Zhang.

*Correspondence and requests for materials should be addressed to Xiaolong Chen (email: chenxl@sustech.edu.cn), Lin Wang (email: iamlwang@njtech.edu.cn) and Xiang-Long Yu (email: yuxl@sustech.edu.cn).

Supplementary Figures

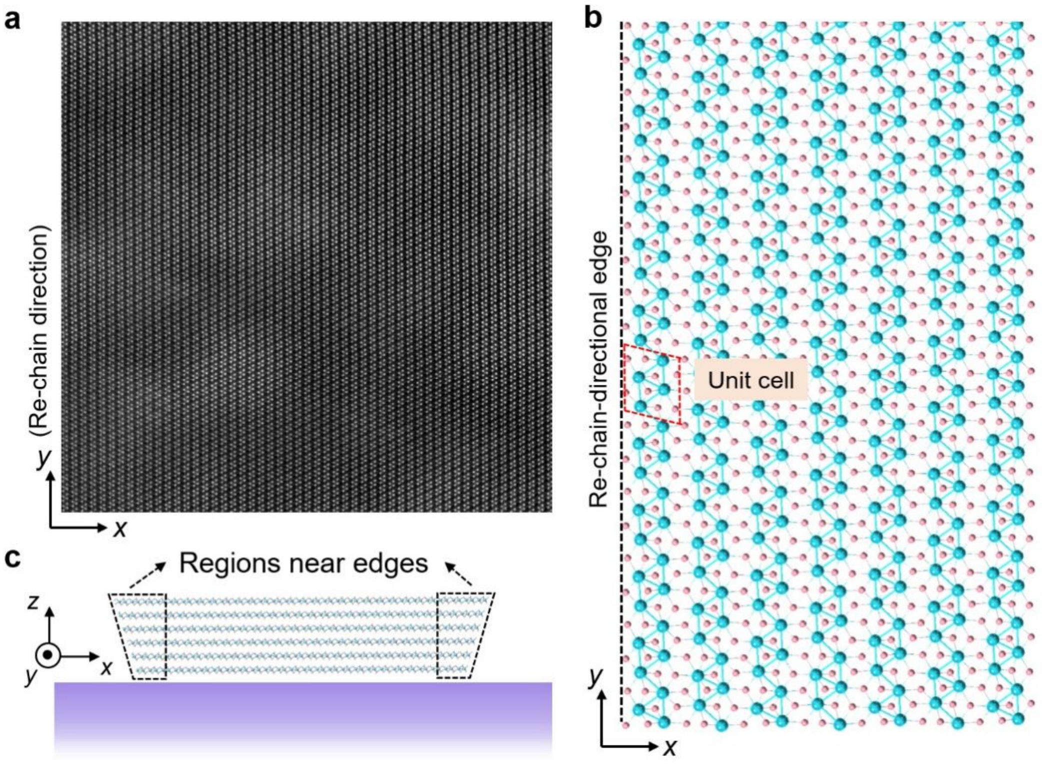

Supplementary Figure 1. **a**, STEM image of ReS_2 crystal (top view). **b**,**c**, Schematic top (**b**) and cross-sectional (**c**) view of the ReS_2 crystalline structure.

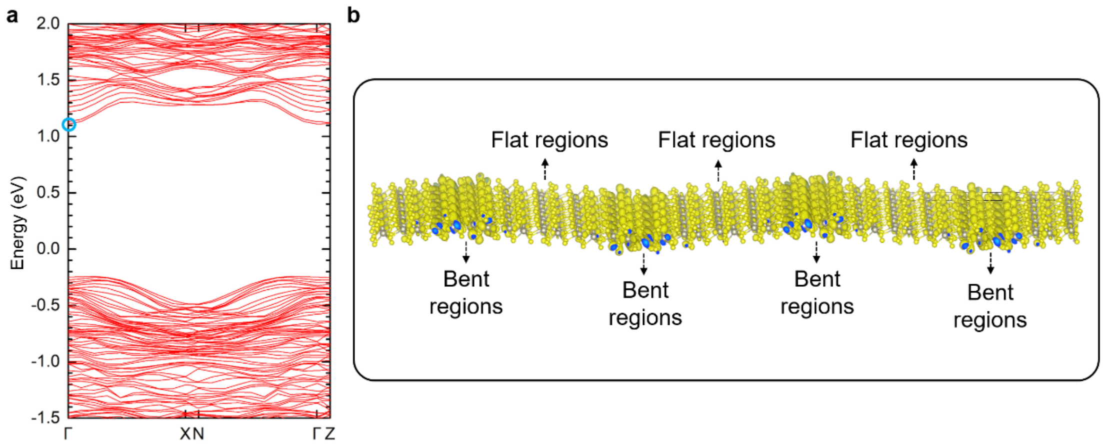

Supplementary Figure 2. **a**, Band structures of bent ReS_2 flake. Strains are along *x*- and *z*-directions **b**, Distribution of electron states at lowest position of conduction band (denoted by the blue circle in **a**) in bent ReS_2 flake.

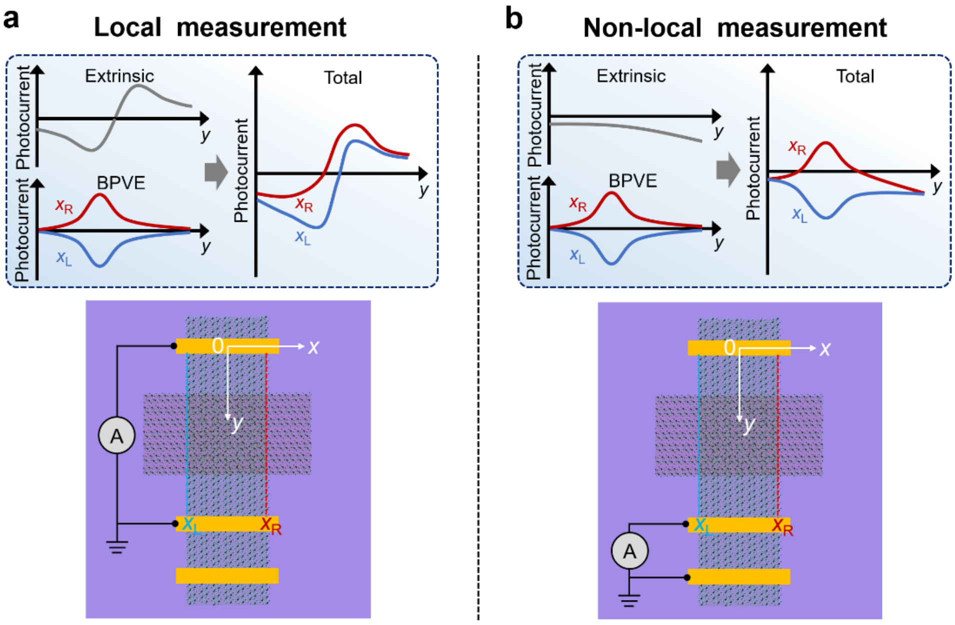

Supplementary Figure 3. Schematics of local (**a**) and non-local (**b**) measurements. The simpler shape of $I_{\mathrm{Extrinsic}}$~y in non-local measurement is preferred to resolve the peak/valley features of BPVE.

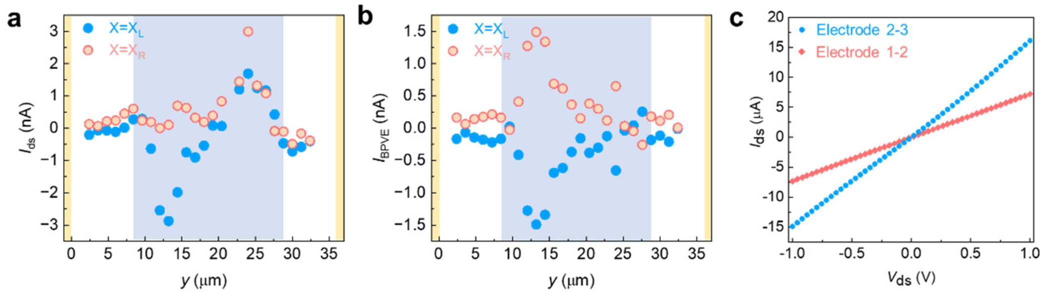

Supplementary Figure 4. **Characterizations of the ReS_2/ReS_2 homostructure device in the main text. a**, Local photocurrents, using electrodes 1 and 2, along left (blue dots) and right (orange dots) edges. Blue shaded region shows the position of edge-embedded region. **b**, Estimated BPVE-induced photocurrents along left and right edges from local photocurrent measurements by $I_{BPVE} = \pm\,(I_{ph}(x_L)-I_{ph}(x_R))/2$. **c**, The linear relationship of current-voltage characteristics indicates that Ohmic contact is achieved between the electrode and the ReS_2 flake.

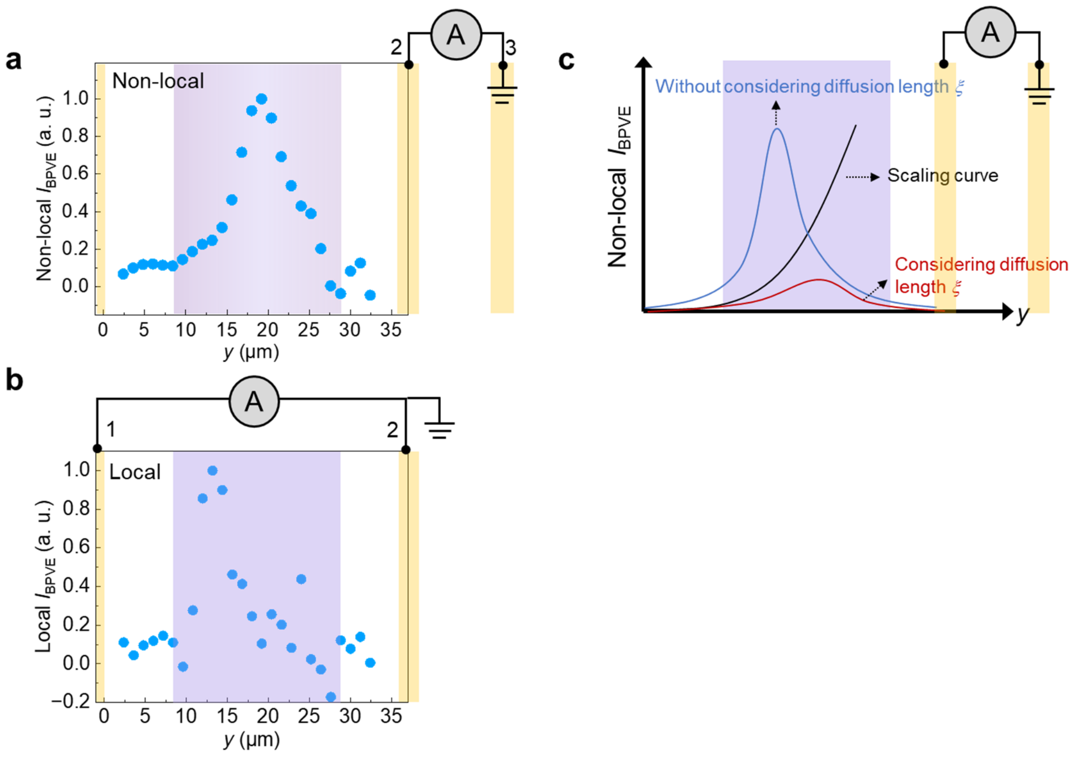

Supplementary Figure 5. **a**, Extracted BPVE-induced photocurrent through non-local measurements. **b**, Extracted BPVE-induced photocurrent through local measurements. **c**, The finite mean diffusion length of carriers ξ might leads to the different peak positions in local and non-local measurements.

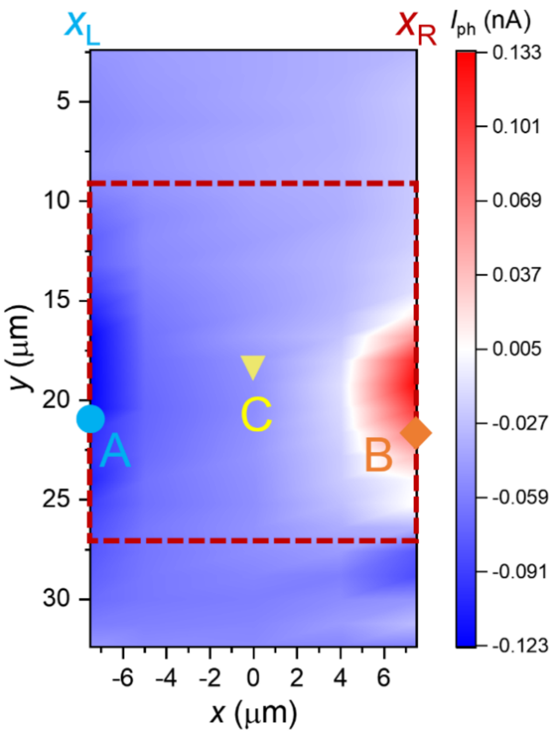

Supplementary Figure 6. Photocurrent mapping of the ReS_2/ReS_2 homostructure device. Red dashed line enclosed area denotes the ReS_2/ReS_2 homostructure region.

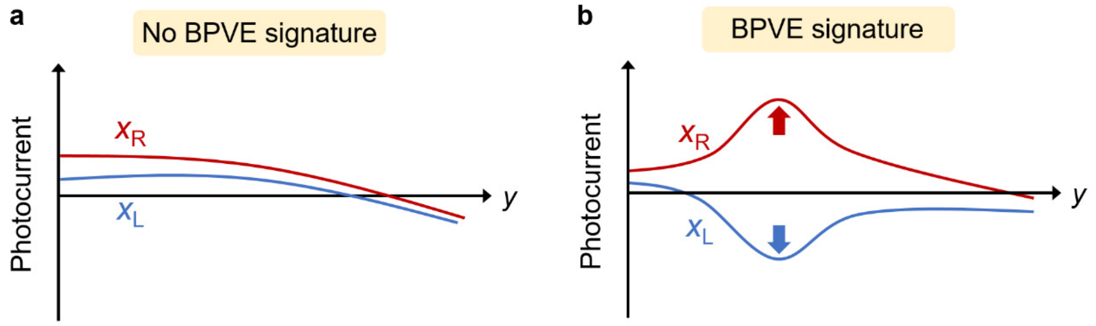

Supplementary Figure 7. **a**, Since left (x_L) and right (x_R) edges are not perfectly the same, difference of photocurrents between left and right edges cannot be simply attributed to BPVE. **b**, Only when remarkable peak and valley features are observed, we are of the opinion that BPVE occurs at two edges.

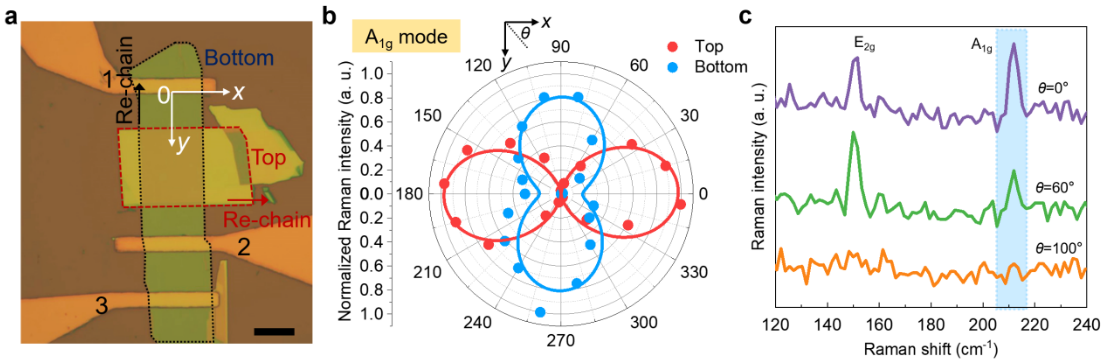

Supplementary Figure 8. **a**, Optical image of the ReS_2/ReS_2 device. **b**,**c**, Polarization-dependent Raman spectra of ReS_2 flakes.

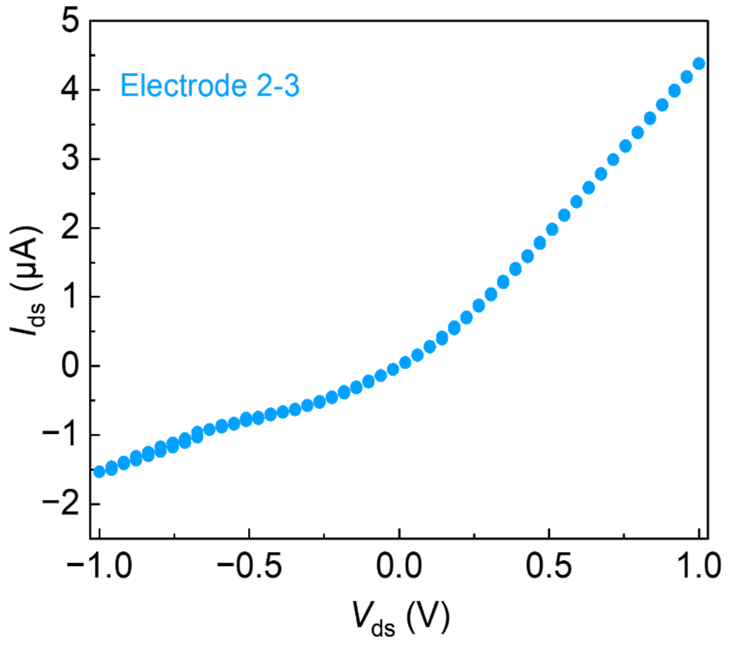

Supplementary Figure 9. Characterizations of the MoS_2/MoS_2 homostructure device in the main text. The nonlinear current-voltage curve probed by electrodes 2 and 3 indicates a Schottky junction exists between the electrode and the MoS_2 flake.

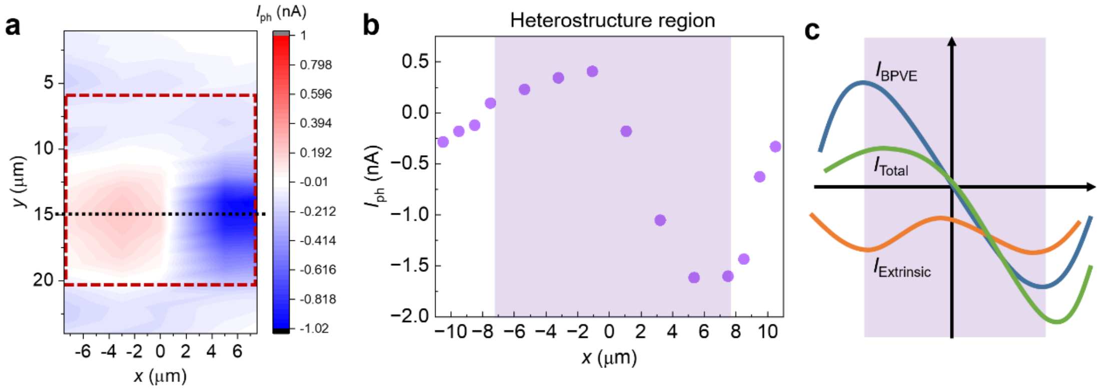

Supplementary Figure 10. **a**, Scanning photocurrent spectroscopy of the WS_2/ReS_2 heterostructure device. **b**, Non-local photocurrent along *x*-direction at *y*=15 μm. **c**, Asymmetric feature of I_{ph} can be attributed to the superposition of I_{BPVE} and $I_{Extrinsic}$.

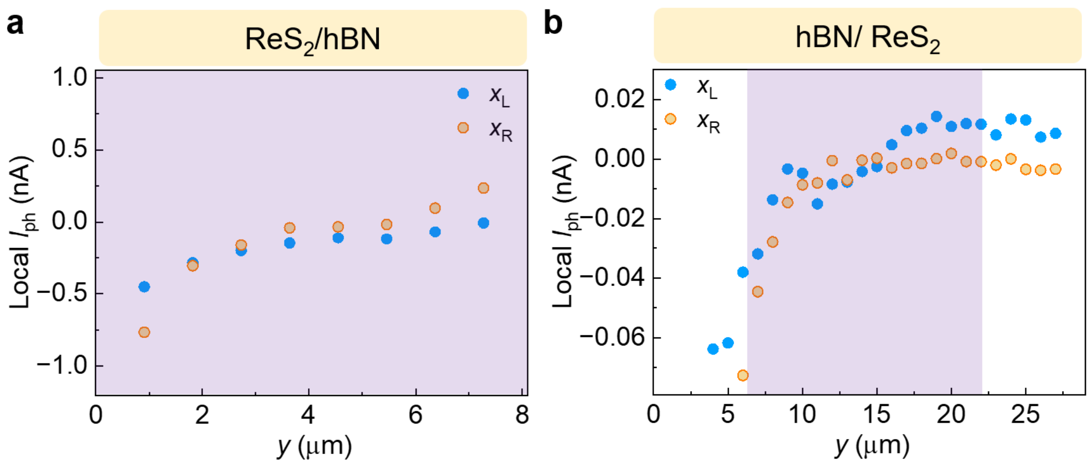

Supplementary Figure 11. Local photocurrent measurements of ReS_2/h-BN (**a**) and h-BN/ReS_2 devices (**b**).

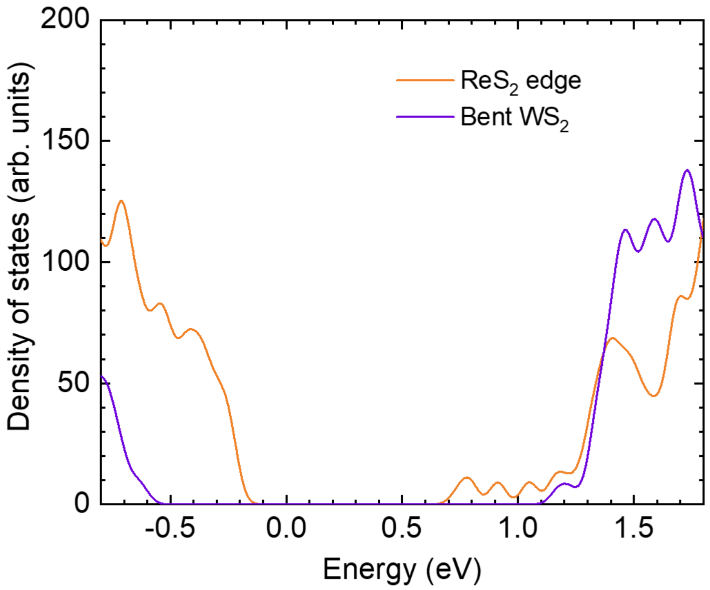

Supplementary Figure 12. The first-principles calculations of density of states of ReS_2 edge and bent WS_2.

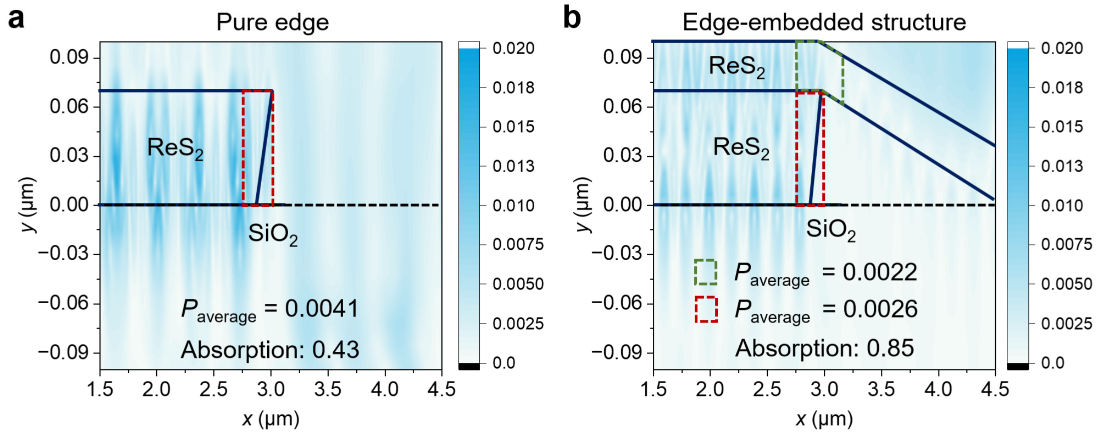

Supplementary Figure 13. **a**,**b**, Power distribution at pure edge (**a**) and edge-embedded structure (**b**).

Supplementary Notes

Supplementary Note 1

Theoretical investigation and origin of bulk photovoltaic effects in edge-embedded structures

Without considering any extrinsic effect, when light (with electric field vector $\vec{E}$ and wave vector $\vec{q}$) shines on materials, a DC current density can be generated which can be expressed as[1, 2]

$$J_{\mathrm{l}}^{\mathrm{DC}} = \sigma_{\mathrm{ljk}}^{(2)}(w,\vec{q})E_{\mathrm{j}}E_{\mathrm{k}}^{*}, \quad (1)$$

where $\sigma_{\mathrm{ljk}}^{(2)}(w,\vec{q})$ is a third rank tensor governed by the geometric symmetry of crystalline structure.

If we only consider the $\vec{q}$-independent and linear polarization-dependent term, we have[1, 2]

$$J_{\mathrm{l}}^{\mathrm{LBPVE}} = \frac{1}{2}\sum_{j,k}\chi_{\mathrm{ljk}}(E_{\mathrm{j}}E_{\mathrm{k}}^{*}+E_{\mathrm{k}}E_{\mathrm{j}}^{*}). \quad (2)$$

Here, $J_{\mathrm{l}}^{\mathrm{LBPVE}}$ is the BPVE-induced current density under linear polarized light along l-direction (l represents x-, y-, or z-direction), and $\chi_{\mathrm{ljk}} = \sigma_{\mathrm{ljk}}^{(2)}(w,0)$. For incident light along z-direction, we have E_{z}=0. Then we have

$$\overrightarrow{J^{\mathrm{LBPVE}}} = \begin{bmatrix} \chi_{\mathrm{xxx}}|E_x|^2 + \chi_{\mathrm{xyy}}|E_y|^2 + \chi_{\mathrm{xxy}}E_{\mathrm{x}}E_{\mathrm{y}}^{*} + \chi_{\mathrm{xxy}}E_yE_x^{*} \\ \chi_{\mathrm{yxx}}|E_x|^2 + \chi_{\mathrm{yyy}}|E_y|^2 + \chi_{\mathrm{yxy}}E_{\mathrm{x}}E_{\mathrm{y}}^{*} + \chi_{\mathrm{yxy}}E_yE_x^{*} \\ \chi_{\mathrm{zxx}}|E_x|^2 + \chi_{\mathrm{zyy}}|E_y|^2 + \chi_{\mathrm{zxy}}E_{\mathrm{x}}E_{\mathrm{y}}^{*} + \chi_{\mathrm{zxy}}E_yE_x^{*} \end{bmatrix}. \quad (3)$$

Because current collection direction is along y-direction, we mainly focus on $J_{\mathrm{y}}^{\mathrm{LBPVE}}$:

$$J_{\mathrm{y}}^{\mathrm{LBPVE}} = \chi_{\mathrm{yxx}}|E_x|^2 + \chi_{\mathrm{yyy}}|E_y|^2 + \chi_{\mathrm{yxy}}E_{\mathrm{x}}E_{\mathrm{y}}^{*} + \chi_{\mathrm{yxy}}E_yE_x^{*}. \quad (4)$$

For materials with inversion symmetry, when we do inversion transformation ($x, y, z\rightarrow$ $-x$, $-y$, $-z$), $\overrightarrow{J^{\mathrm{LBPVE}}}(x,y,z) = -\overrightarrow{J^{\mathrm{LBPVE}}}(-x,-y,-z)$. χ_{ljk} must be zero. Hence, no BPVE

effect can be observed in systems with inversion symmetry. In the following section, we will discuss three situations: 1) pure edges with mirror symmetry; 2) pure ReS_2 edges; 3) ReS_2 edge-embedded structures.

1) Pure edges with mirror symmetry

We take <100>-directional edge of WTe_2 as an example[3]. A mirror symmetry plane M_a exists in <100>-directional edge. Under mirror symmetry (x, y, $z \rightarrow x$, $-y$, z), $J_y^{LBPVE}(x,y,z) = -J_y^{LBPVE}(x,-y,z)$, which makes $\chi_{yxx} = \chi_{yyy} = 0$. For linear polarized light, $\vec{E} = [E_0 \cos\alpha, E_0 \sin\alpha, 0]$ where α is the angle between x-direction and light polarization direction. Then we have

$$J_y^{LBPVE} = \chi_{yxy} E_0^2 \sin 2\alpha. \quad (5)$$

Hence, from the theoretical perspective, there should be a y-directional BPVE current along the edge when light polarization is not along x- and y- directions. The BPVE current vanishes when light is polarized along x- and y- directions. However, previous experiments on WTe_2 <100>-edges (*Nat. Commun.* 10, 5736, 2019) did not observe detectable J_y^{LBPVE} [3]. This is probably due to the small value of χ_{yxy} in WTe_2.

2) Pure ReS_2 edges

We will focus on edges along Re-chain direction. As shown in Supplementary Fig. 1, no mirror symmetry or rotation symmetry exist in ReS_2 edges. Hence, J_y^{LBPVE} along ReS_2 edge can be expressed as

$$J_y^{LBPVE} = 2\chi_{yxx} E_0^2 \cos^2\alpha + 2\chi_{yyy} E_0^2 \sin^2\alpha + 2\chi_{yxy} E_0^2 \sin 2\alpha. \quad (6)$$

We rewrite equation (6) to

$$J_y^{LBPVE} = (\chi_{yxx} + \chi_{yyy}) E_0^2 + (\chi_{yxx} - \chi_{yyy}) E_0^2 \cos 2\alpha + 2\chi_{yxy} E_0^2 \sin 2\alpha. \quad (7)$$

Here, the first term $(\chi_{yxx} + \chi_{yyy}) E_0^2$ is polarization-independent, and second and third terms are polarization-dependent. From the theoretical perspective, there should be a y-directional BPVE photocurrent along pure ReS_2 edges. However, we did not observe

obvious signal along ReS_2 edges experimentally, probably due to the small value of χ_{ljk}. On the other hand, the inversion symmetry is still approximately preserved in unit cell at edges (see Supplementary Fig. 1b). It could probably significantly reduce the BPVE signal.

3) ReS_2 edge-embedded structures

In ReS_2 edge-embedded structures, top ReS_2 layers are coupled with bottom ReS_2 edges. Regions near ReS_2 edges should show distinct properties from inner regions. Thus, we treat edge-embedded regions as a whole (see Fig. 1d in main text). Besides, strains also exist in edge-embedded structures in *x*- and *z*- directions. Hence, compared with pure ReS_2 edges, the geometric symmetry of ReS_2 edge-embedded structures is further lowered. The BPVE-induced photocurrent can also be expressed as $J_y^{LBPVE} = (\chi'_{yxx} + \chi'_{yyy})E_0^2 + (\chi'_{yxx} - \chi'_{yyy})E_0^2 \cos 2\alpha + 2\chi'_{yxy} E_0^2 \sin 2\alpha$ or $J_y^{LBPVE} = D + C\cos(2\alpha + \varphi)$, where $D = (\chi'_{yxx} + \chi'_{yyy})E_0^2$ and $C = \sqrt{(\chi'_{yxx} - \chi'_{yyy})^2 + 4\chi'^2_{yxy}} E_0^2$, and φ is the phase. However, the second-order nonlinear DC tensor χ'_{ijk} of edge-embedded structures should be different from that of pure edges χ_{ijk}, since it depends not only on the type of materials but also on the geometry. The lowered symmetry and different geometry configuration of ReS_2 edge-embedded structures could probably enhance the value of χ'_{ijk}.

On the other hand, charge transfers inside ReS_2 edge-embedded structures can further enhance the BPVE photocurrent along edges. To support this, we have performed the first-principles calculations on band structures of ReS_2, ReS_2 edges, and strained ReS_2. The band structure and density of states (DOS) of a ReS_2 ribbon with edges along Re-chain direction are calculated (see Fig. 4f-h in the main text). An energy band, generated by edge states, appears inside the band gap of ReS_2. For top ReS_2 layers, there are strains along *x*- and *z*- directions near edge regions. Hence, we further calculated the

band structure of bent ReS_2. As shown in Supplementary Fig.2, at lowest position of conduction band (Γ point, marked by the blue circle), electron states are mostly distributed near bent regions. This indicates that photo-carriers generated in top ReS_2 layers tend to gather in bent regions.

Finally, we compared the DOS of ReS_2 edges and bent ReS_2 near the band edge. As shown in Fig. 4h in the main text, charge transfers are enabled from top bent ReS_2 to bottom ReS_2 edges. Although electrodes are fabricated on bottom ReS_2, charge transfers allow us to detect BPVE-generated carriers from top ReS_2 region.

Supplementary Note 2

Local and non-local photocurrent measurement

As schematically shown in Supplementary Fig. 3, the photocurrents from extrinsic photovoltaic effect $I_{\mathrm{Extrinsic}}$ show different shapes for local and non-local measurements. For local measurement, the target channel is located between drain and source electrodes. Due to the symmetry of device, $I_{\mathrm{Extrinsic}}$ will change direction and show many peak/valley features when laser spot moves from drain to source (see Supplementary Fig. 3a). For non-local measurement, the target channel is located outside drain and source electrodes. Photon-generated carriers will diffuse to electrodes. Closer to electrodes, easier for carriers to reach electrodes. Thus, $|I_{\mathrm{Extrinsic}}|$ will monotonously increase when approaching electrodes (see Supplementary Fig. 3b and Supplementary Fig. 4). The simpler shape of $I_{\mathrm{Extrinsic}}$~y in non-local measurement is preferred to resolve the peak/valley features of BPVE.

The peaks of BPVE-induced photocurrents are indeed at different positions for non-

local and local measurements as shown in Supplementary Fig. 5. We attribute this phenomenon to following reasons. Firstly, for local measurements, the electrodes 1 and 2 are not perfectly symmetric. As a result, the peak may not locate in the middle position of channel (see Supplementary Fig. 5). Secondly, for non-local measurements, photocurrents are generated when photo-excited carriers diffuse to electrode 2. In real situations, the mean diffusion length ξ is finite. Thus, nonlocal photocurrent also strongly depends on the distance between laser spot and electrode 2. Shorter distance will result in a larger photocurrent. We can introduce a scaling curve $l(y)$ that describes the diffusion characteristics of carriers. Based on conventional semiconductor theory, $l(y)=\exp(-y/\xi)$. If we assume the non-local photocurrent with and without considering ξ to be $f(y)$ and $g(y)$, respectively, we have $f(y)=\exp(-y/\xi)g(y)$. The scaling term $\exp(-y/\xi)$ will result in a shift of photocurrent peak towards electrode 2 (see Supplementary Fig. 5).

Supplementary References

1. Jiang, J. et al. Flexo-photovoltaic effect in MoS_2. *Nat. Nanotechnol.* **16**, 894-901 (2021).
2. Quereda, J. et al. Symmetry regimes for circular photocurrents in monolayer $MoSe_2$. *Nat. Commun.* **9**, 3346 (2018).
3. Wang, Q. et al. Robust edge photocurrent response on layered type II Weyl semimetal WTe_2. *Nat. Commun.* **10**, 5736 (2019).
